# Real-World Analysis of Metastatic Renal Cell Carcinoma Patients Treated with Pembrolizumab Plus Axitinib: Evidence from the Campania Oncology Network

**DOI:** 10.3390/curroncol33060325

**Published:** 2026-05-30

**Authors:** Marilena Di Napoli, Elisabetta Coppola, Carmine D’Aniello, Sarah Scagliarini, Carlo Buonerba, Andrea Muto, Luigi Formisano, Francesco Sabbatino, Davide Bosso, Sabrina Rossetti, Lorenzo Lobianco, Rosa Tambaro, Fabrizio Di Costanzo, Pasquale Rescigno, Carmela Pisano, Sabrina Chiara Cecere, Anna Passarelli, Jole Ventriglia, Gabriele Calvanese, Maria Rosaria Lamia, Erica Perri, Roberto Contieri, Dario Franzese, Maria Adelina Simeoni, Caterina Mariarosaria Giorgio, Florinda Feroce, Salvatore Stilo, Giovanni Pacifico, Giuseppina Canciello, Sandro Pignata

**Affiliations:** 1 Department of Urology and Gynecology, Istituto Nazionale Tumori di Napoli, IRCCS ‘G. Pascale’, 80131 Naples, Italy; elisabetta.coppola@istitutotumori.na.it (E.C.); s.rossetti@istitutotumori.na.it (S.R.); lorenzo.lobianco@istitutotumori.na.it (L.L.); r.tambaro@istitutotumori.na.it (R.T.); c.pisano@istitutotumori.na.it (C.P.); s.cecere@istitutotumori.na.it (S.C.C.); anna.passarelli@istitutotumori.na.it (A.P.); j.ventriglia@istitutotumori.na.it (J.V.); gabriele.calvanese@unimi.it (G.C.); mariarosaria.lamia@unina.it (M.R.L.); erica.perri@studenti.unicampania.it (E.P.); giovanni.pacifico@studenti.unicampania.it (G.P.); giuseppina.canciello@istitutotumori.na.it (G.C.); s.pignata@istitutotumori.na.it (S.P.); 2 Division of Medical Oncology, AORN dei Colli-Monaldi Hospital, 80131 Naples, Italy; carmine.daniello@hotmail.it; 3 Department of Medical Oncology, AORN “A. Cardarelli”, 80131 Naples, Italy; sarahscagliarini@gmail.com; 4 Oncology Unit, “Andrea Tortora” Hospital, 84016 Pagani, Italy; spec.buonerbac@aslsalerno.it; 5 Division of Medical Oncology, “San Giuseppe Moscati” Hospital, 83100 Avellino, Italy; mutoeco@libero.it; 6 Department of Clinical Medicine and Surgery, University of Naples “Federico II”, 80138 Napoli, Italy; luigi.formisano1@unina.it; 7 Oncology Department, University Hospital “San Giovanni di Dio e Ruggi d’Aragona”, 84131 Salerno, Italy; sabbatinof@gmail.com; 8 Oncology Unit, Ospedale del Mare, 80147 Naples, Italy; davidebosso84@gmail.com; 9 Translational and Clinical Research Institute, Newcastle University Centre for Cancer, Newcastle Upon Tyne NE2 4HH, UK; fabrizio.dicostanzo@unina.it (F.D.C.); pasquale.rescigno@newcastle.ac.uk (P.R.); 10 Department of Urology, Istituto Nazionale Tumori di Napoli, IRCCS ‘G. Pascale’, 80131 Naples, Italy; roberto.contieri@istitutotumori.na.it (R.C.); dario.franzese@istitutotumori.na.it (D.F.); 11 Nephrology and Dialysis Unite, Department of Translation Medicine Medical Sciences, University of Campania “Luigi Vanvitelli”, 80138 Naples, Italy; mariadelina.simeoni@unicampania.it; 12 Dermatology Unit, Department of Mental and Physical Health and Preventive Medicine, University of Campania Luigi Vanvitelli, 81100 Naples, Italy; caterinagiorgio80@gmail.com; 13 Department of Pathology, Istituto Nazionale Tumori di Napoli, IRCCS ‘G. Pascale’, 80131 Naples, Italy; f.feroce@istitutotumori.na.it; 14 Interventional Radiology Unit, Istituto Nazionale Tumori di Napoli, IRCCS ‘G. Pascale’, 80131 Naples, Italy; salvatore.stilo@istitutotumori.na.it

**Keywords:** metastatic renal cell carcinoma, immunotherapy, TKI, real-world data, real-world evidence, pembrolizumab, axitinib

## Abstract

**Background**: Immune checkpoint inhibitor–tyrosine kinase inhibitor combinations represent a standard first-line option for metastatic renal cell carcinoma (mRCC). However, patients enrolled in pivotal trials often differ from those treated in routine practice. We report real-world outcomes of pembrolizumab plus axitinib within the Campania Oncology Network. **Methods**: We conducted a multicenter retrospective study including consecutive treatment-naïve mRCC patients who received first-line pembrolizumab plus axitinib between January 2021 and November 2023 across eight regional centers. The primary endpoints were progression-free survival (PFS) and overall survival (OS); secondary endpoints included objective response rate (ORR) and safety. **Results**: A total of 117 patients were included. IMDC risk was favorable in 19.6%, intermediate/poor in 65%, and unknown in 15.4%. Median age was 59 years, and 53.8% had ECOG performance status ≥ 1. Clear-cell histology accounted for 87.2% of cases; brain metastases were present in 36%. After a median follow-up of 12.8 months, median PFS was 15.1 months (95% CI 9.6–NR), and median OS was not reached. ORR was 27.3%, with a disease control rate of 79.5%; patients with non-clear-cell histology showed an ORR of 41.7%. Disease progression was the main cause of treatment discontinuation (49%), while adverse events (AEs) led to discontinuation in 6% of cases. Grade ≥3 AEs occurred in 14% of patients. Most toxicities were grade 1–2, including diarrhea (23.9%), asthenia (18%), hypothyroidism (12.8%), and hypertension (9.4%). Grade 1–2 AEs were significantly more frequent in females compared with males (57.6% vs. 35.7%, *p* = 0.05). **Conclusions**: In this consecutive regional cohort, pembrolizumab plus axitinib showed clinically relevant disease control, although ORR was lower than in pivotal trials. Performance status emerged as a key prognostic factor. Real-world data from oncology networks may support personalized first-line treatment decisions in mRCC.

## Simple Summary

Kidney cancer may spread to other organs and require systemic treatment. The combination of pembrolizumab and axitinib is an established first-line treatment option, but patients included in clinical trials may differ from those treated in everyday practice. We analyzed consecutive patients with metastatic kidney cancer treated with pembrolizumab plus axitinib within the Campania Oncology Network. The treatment showed clinically relevant disease control in a real-world regional population, although the response rate was lower than that reported in pivotal trials. Performance status was the main factor associated with patient outcomes. These findings support the value of regional oncology networks for monitoring real-world treatment results and may help clinicians interpret trial evidence in routine care.

## 1. Introduction

Renal cell carcinoma (RCC) represents the 14th most frequently diagnosed malignancy worldwide [1,2]. Although its incidence has steadily increased over the past decades, overall mortality has declined—likely due to the more frequent detection of early-stage tumors through abdominal imaging, as well as to remarkable advances in systemic therapies [3]. More specifically, the therapeutic landscape of RCC has been reshaped by the introduction of vascular endothelial growth factor receptor (VEGFR) tyrosine kinase inhibitors (TKIs) and, recently, the immune checkpoint inhibitors (ICIs), reinforcing treatment strategies for both the localized and advanced disease. In both settings, the new approaches demonstrated significant improvements in progression-free survival (PFS) and overall survival (OS) endpoints compared with single-agent TKI therapy, primarily sunitinib, in several large randomized phase III studies, including the CheckMate 214, KEYNOTE-426, CheckMate 9ER, and CLEAR trials [3,4,5,6,7,8]. On the basis of these data, current clinical guidelines [3,4,5,6,7,8] recommend, as first-line treatment for advanced RCC, either dual immune checkpoint blockade with ipilimumab plus nivolumab in patients with intermediate- or poor-risk disease according to the International Metastatic RCC Database Consortium (IMDC) criteria, or a combination of an ICI with a TKI—namely pembrolizumab plus axitinib, nivolumab plus cabozantinib, or pembrolizumab plus lenvatinib—across all IMDC risk categories. More recently, the COSMIC-313 study demonstrated that the triplet regimen combining ipilimumab, nivolumab, and cabozantinib significantly improved PFS in treatment-naïve patients with metastatic intermediate- or poor-risk RCC, although at the expense of increased toxicity [9]. However, in the absence of mature overall survival data, this triplet strategy is not currently recommended [3].

Despite the availability of multiple effective therapeutic options, treatment selection in advanced RCC remains challenging. No prospective head-to-head trials have directly compared the approved immune-based combinations, and validated predictive biomarkers are still lacking. Therefore, in routine clinical practice, treatment decisions continue to rely largely on clinical parameters, including tumor burden, performance status, comorbidities, IMDC risk group, histology, metastatic pattern, or patient preference. Moreover, the IMDC prognostic score, originally developed in the TKI era, may not fully capture the biological heterogeneity and prognostic complexity of patients treated in the contemporary immunotherapy-based setting [10].

In this context, RCC represents a clinically relevant model for integrating real-world and translational oncology approaches. Structured real-world datasets generated within oncology networks may complement randomized trial evidence by capturing patient heterogeneity, treatment patterns, safety, and outcomes in populations that are often underrepresented in clinical trials. Such datasets may also provide a foundation for future integration of clinical, imaging, pathological, and molecular information to support more individualized treatment strategies.

Importantly, patients enrolled in pivotal clinical trials do not always reflect the broader population encountered in daily clinical practice, as trial participants typically have better performance status and fewer comorbidities compared with real-world patients [11,12]. Consequently, the efficacy and safety outcomes observed in controlled trial settings may not be entirely generalizable to unselected populations. For this reason, international regulatory and scientific authorities strongly encourage the systematic collection and analysis of real-world data (RWD) to generate complementary real-world evidence (RWE) [13]. In this scenario, several studies represent an important example of international real-world evidence reporting and serve as a relevant reference for the interpretation of outcomes observed in RCTs. Among these, the ARON-1 study provides insightful data on patients treated with pembrolizumab plus axitinib outside the context of pivotal clinical trials [14]. Similarly, Regional Oncology Networks represent a valuable infrastructure for the generation of high-quality RWD and the promotion of RWE to support evidence-based clinical decision-making [15].

In 2016, the Campania region in Italy established the Campania Oncology Network (*Rete Oncologica Campana*, *ROC*) [16], with the aim of standardizing oncologic care and improving access to high-quality cancer treatment across the region. The ROC integrates multiple oncology centers into structured Multidisciplinary Oncology Groups (*Gruppi Oncologici Multidisciplinari*, *GOM*), where treatment decisions are systematically discussed; a dedicated digital platform records these discussions, thereby contributing to the structured generation of RWD [16]. Furthermore, through the ONCOCAMP protocol, the network systematically analyzes the processes and outcomes of consecutive patients treated within the region across 31 different cancer entities. Among its objectives, the ROC seeks to evaluate and compare available therapeutic options in clinical scenarios where multiple treatment strategies are feasible.

Accordingly, to present our regional experience and to compare real-world effectiveness, safety, and tolerability outcomes with those reported in registrational trials and in the aforementioned retrospective studies, we analyzed data from consecutive patients treated with pembrolizumab plus axitinib within the GOMs of the ROC.

## 2. Study Population

This study was conducted within the Campania Oncology Network (Rete Oncologica Campana, ROC) under the ONCOCAMP project, promoted by the Istituto Nazionale Tumori—IRCCS—Fondazione “G. Pascale” in Naples, Italy. The study protocol was approved by the local Ethics Committee and was conducted in accordance with the revised Declaration of Helsinki (52nd WMA General Assembly, Edinburgh, Scotland, October 2000).

We retrospectively collected data from consecutive patients aged ≥18 years with a histologically confirmed diagnosis of metastatic renal cell carcinoma who received first-line treatment with pembrolizumab plus axitinib between January 2021 and November 2023 at one of the ROC centers. All patients were registered on the dedicated digital platform of the Campania Oncology Network and were included in the present analysis.

The collection and analysis of data from patients treated with other immune-based combinations within the network are ongoing and will be reported separately once a comparable duration of follow-up is reached. Pembrolizumab plus axitinib was the first immune-based combination reimbursed in the Campania region, which explains its earlier and broader adoption in this setting.

### 2.1. Evaluation of Outcomes

Tumor response was assessed according to the Response Evaluation Criteria in Solid Tumors (RECIST), version 1.1, and categorized as complete response (CR), partial response (PR), stable disease (SD), or progressive disease (PD).

The following clinical parameters were collected: baseline comorbidities, causes of treatment discontinuation, progression-free survival (PFS), overall survival (OS), objective response rate (ORR), and treatment-related toxicities. ORR was defined as the proportion of patients achieving either CR or PR. After initiation of pembrolizumab plus axitinib, patients were routinely assessed before each pembrolizumab cycle, including clinical examination, evaluation of performance status, concomitant medications, and toxicity review. Laboratory tests, including complete blood count, renal and liver function tests, thyroid function, electrolytes, and urinalysis/proteinuria assessment, were generally performed at baseline and at regular intervals during treatment, according to institutional practice and clinical need. Radiologic tumor assessment was performed by contrast-enhanced CT of the chest, abdomen, and pelvis every 8–12 weeks during treatment, or earlier in case of clinical deterioration. Additional imaging, like either brain MRI/CT or bone imaging, was performed at baseline and when clinically indicated, including for follow-up of known lesions. Responses were retrospectively classified according to RECIST v1.1 based on available radiology reports and imaging review.

Treatment-related adverse events were retrospectively retrieved from medical records, treatment charts, laboratory results, and clinical notes. Adverse events were graded according to the National Cancer Institute Common Terminology Criteria for Adverse Events (CTCAE), version 4.0.

### 2.2. Statistical Analysis

Demographic and clinical characteristics are summarized using descriptive statistics. Categorical variables are reported as frequencies and percentages, while continuous variables are expressed as median and interquartile range (IQR).

The primary endpoints of the study were progression-free survival (PFS) and overall survival (OS). Secondary endpoints included objective response rate (ORR) and safety. PFS was defined as the time from initiation of pembrolizumab plus axitinib treatment to documented disease progression or death from any cause, whichever occurred first. OS was defined as the time from treatment initiation to death from any cause or censoring at the date of last follow-up. Survival distributions were estimated using the Kaplan–Meier method, and differences between survival curves were assessed using the log-rank test. ORR was reported as a percentage. Adjusted hazard ratios (HRs) and corresponding 95% confidence intervals (CIs) were estimated using multivariable Cox proportional hazards regression models, adjusting for baseline clinical characteristics, including ECOG performance status and selected clinically relevant baseline covariates defined a priori: age, sex, IMDC risk score, and number of metastatic sites. The proportional hazards assumption was assessed using Schoenfeld residuals. Missing baseline covariate data were not imputed. For multivariable Cox proportional hazards regression models, complete-case analysis was performed; therefore, patients with missing values in any covariate included in a given model were excluded from that model.

All statistical analyses were performed using R software (version 4.3.3).

## 3. Results

Between January 2021 and November 2023, a total of 117 patients were treated across eight centers within the ROC. All patients received at least one dose of pembrolizumab plus axitinib. Baseline demographic and disease characteristics are summarized in Table 1.

Most patients had clear-cell histology (102/117, 87.2%), while non-clear-cell subtypes included papillary, chromophobe, and mixed histologies; sarcomatoid features were documented in 4 patients (3.4%). Overall, 79 patients (67.5%) presented with de novo metastatic disease, and 53 patients (45.3%) had undergone previous nephrectomy. Baseline disease burden in our real-world cohort was heterogeneous, with frequent involvement of the brain (36.8%), lung (35.0%), bone (30.8%), and lymph nodes (29.1%); less frequent metastatic sites included the adrenal glands, liver, pancreas, peritoneum, spleen, and rectum. According to IMDC criteria, 19.6% of patients were classified as favorable risk, 47.0% as intermediate risk, and 18.0% as poor risk, while 15.4% were unknown. ECOG performance status was 0 in 30.8%, 1 in 47.9%, ≥2 in 5.9%, and unknown in 15.4% of patients.

### 3.1. Efficacy Endpoints

**Figure 1:**
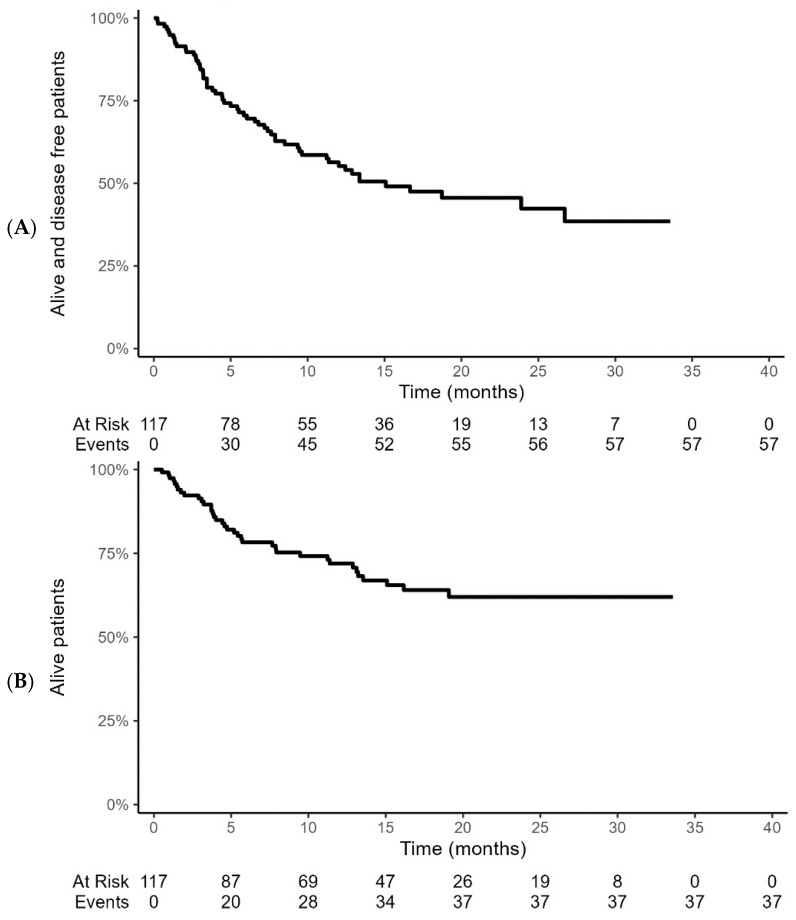
Progression-Free Survival (**A**) and Overall Survival (**B**) in the overall population.

Overall, 57 patients (48.7%) experienced disease progression or death. The median progression-free survival for the entire cohort was 15.1 months (95% CI: 9.63–NA) (Figure 1A), with a 12-month progression-free probability of 56.3% (95% CI: 47.6–66.7%). After a median follow-up of 12.8 months (range 0.5–33.6), the median OS was not reached. Thirty-seven deaths (32%) were recorded (Figure 1B), and the estimated 12-month survival rate was 72% (95% CI: 63.9–81.1%).

According to RECIST v1.1 criteria, 2 patients achieved complete response (CR) and 30 achieved partial response (PR), resulting in an objective response rate (ORR) of 27.3% (95% CI: 16.0–32.0%); the disease control rate (DCR) was 79.5% (95% CI: 72.2–86.8%).

The primary reason for treatment discontinuation was disease progression. Seven patients (6.0%) discontinued therapy due to adverse events, and one patient stopped treatment by personal choice.

**Figure 2:**
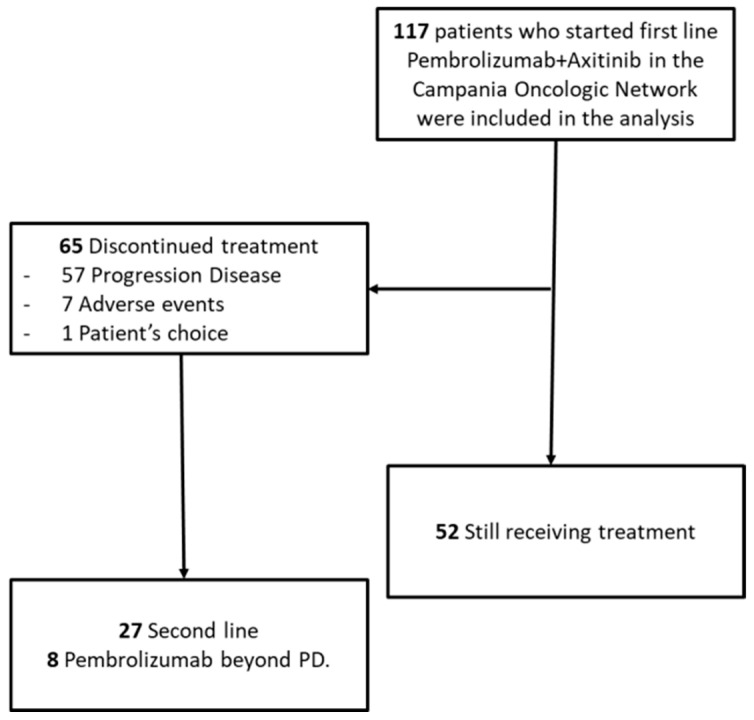
Patient flow diagram.

Among the 65 patients (55%) who discontinued pembrolizumab plus axitinib, 27 (41%) received subsequent anticancer therapy—most commonly cabozantinib. Eight patients (12.3%) continued pembrolizumab beyond radiological progression (Figure 2); for these, the median treatment duration was 10.87 months (IQR 3.81–18.86).

At the time of analysis, 52 patients (44.4%) were still receiving treatment.

### 3.2. Subgroup Analysis by Histology

In patients with clear-cell RCC (n = 102), median PFS was 12.9 months (95% CI: 8.5–NA), and median OS was not reached. ORR was 26.5% (95% CI: 17.9–35.0%), DCR was 78.4% (95% CI: 70.4–86.4%), and the PD rate was 21.6%.

In patients with non-clear-cell RCC (n = 12), median PFS was 16.7 months (95% CI: 7.6–NA), and median OS was not reached. ORR was 41.7% (95% CI: 13.8–69.5%), DCR was 83.3% (95% CI: 62.1–100%), and the PD rate was 16.7% (Table 2 and Table S1).

### 3.3. Outcomes According to Brain Metastatic Status

Given the unexpectedly high proportion of patients with brain metastases in the study population, an exploratory subgroup analysis was performed according to brain metastatic status. Overall, 43 patients had documented brain metastases at baseline, while 74 patients had no brain metastases. Among patients without brain metastases, median PFS was 18.7 months (95% CI 9.36–NA), compared with 13.4 months (95% CI 6.57–NA) among patients with brain metastases; this difference was not statistically significant (log-rank *p* = 0.60). Median OS was not reached in either subgroup, with no statistically significant difference between groups (log-rank *p* = 0.63). ORR was 28.4% in patients without brain metastases and 25.6% in those with brain metastases, while DCR was 78.4% and 81.4%, respectively. No statistically significant differences in ORR or DCR were observed according to brain metastatic status.

### 3.4. Prognostic Factors

Univariate analysis (Table S1) identified PS-ECOG as a strong predictor of both PFS and OS. Patients with PS-ECOG ≥ 2 had a median PFS of 4.4 months and a median OS of 9.5 months, whereas those with PS-ECOG 0–1 had a median PFS of 13.4 months and a median OS not reached (PFS *p* = 0.001; OS *p* = 0.02).

The IMDC risk group showed numerically different PFS estimates across categories, although the association did not reach statistical significance (log-rank *p* = 0.07). Median PFS was not reached in patients with favorable risk (95% CI: 23.9–NA), compared with 16.7 months (95% CI: 9.4–NA) and 12.9 months (95% CI: 4.4–NA) in patients with intermediate and poor risk, respectively. Gender, age, histology, and number of metastatic sites at diagnosis were not significantly associated with PFS or OS.

In multivariate Cox regression models (Tables S2 and S3), adjusting for age, sex, IMDC risk score, and number of metastatic sites, PS-ECOG ≥ 2 remained independently associated with increased risk of progression (HR 5.2, 95% CI 1.8–14.4; *p* = 0.001) and death (HR 5.9, 95% CI 1.7–20.4; *p* = 0.005). Other covariates did not significantly impact survival outcomes; event counts by covariate category are reported in Tables S2 and S3.

### 3.5. Safety

Fifty-three patients (45.3%) experienced at least one treatment-related AE, and 16 patients (14%) developed grade ≥3 toxicity (Table 3). The most frequent AEs were diarrhea (23.9%), asthenia (18.0%), hypothyroidism (12.8%), hypertension (9.4%), and mucosal inflammation (7.7%).

AE-related discontinuations occurred in seven patients (6%). Severe toxicities included three cases of diarrhea, one hand-foot syndrome, one pulmonary embolism, one autoimmune pneumonitis, and one case of ulcerative colitis that led to the patient’s exitus.

When stratified by gender, all-grade AEs were reported in 60.6% of females (20/33) and 39.3% of males (33/84). Similarly, grade 1–2 toxicity was more frequent in females (57.6%) compared with males (35.7%) (*p* = 0.05). No other statistically significant difference was observed for grade 3–4 toxicity or overall AE frequency (*p* = 0.06).

## 4. Discussion

In the Campania Region, standardized mortality ratios have been reported as higher than the national average for several cancers [14]. These epidemiological data prompted the development of a regional oncology network aimed at improving care organization and systematically monitoring treatment outcomes. Approximately 1100 new diagnoses of renal cancer occur annually in the region, with 1024 patients undergoing nephrectomy, while the remaining cases present with primary metastatic disease.

Immune-based combinations—either dual ICI or ICI plus TKI—are currently the standard first-line treatment for mRCC; however, no randomized head-to-head trials comparing the available regimens have been conducted. Pembrolizumab plus axitinib was the first ICI-TKI combination approved by the FDA and EMA, based on the KEYNOTE-426 trial, and the first reimbursed in the Campania region. In the absence of predictive biomarkers or comparative trials, treatment decisions rely on tumor burden, performance status, IMDC risk group, comorbidities, and patient preference. In this context, real-world data are crucial for refining treatment strategies.

In our consecutive real-world cohort of 117 patients, median PFS was 15.1 months, in line with the first interim analysis of KEYNOTE-426 and with the ARON-1 study, which reported real-world outcomes of first-line immunotherapy-based combinations in mRCC (Table 4). However, ORR was markedly lower than in KEYNOTE-426 (27.3% vs. 59.3%) [17,18]; this discrepancy may be explained by several non-mutually exclusive factors.

First, our cohort included a less selected population, characterized by a high proportion of de novo metastatic disease, a relatively low rate of prior nephrectomy (45.3% vs. 82.6% in KEYNOTE-426), and a high prevalence of brain metastases (36.8%), all suggesting a greater baseline disease burden. Second, tumor assessments were not centrally reviewed and were performed according to local clinical practice, with variability in timing and documentation; as a result, partial responses may have been under-recorded when tumor shrinkage was not formally confirmed or when radiology reports did not allow strict retrospective RECIST categorization. Finally, despite the low ORR, the high disease-control rate (79.5%) suggests that a substantial proportion of patients derived clinical benefit through prolonged stable disease rather than objective tumor shrinkage. This may partly explain why the median PFS was not proportionally reduced. Overall, these factors limit direct cross-trial comparisons and support a cautious interpretation of ORR in this retrospective real-world cohort.

Despite a small cohort of 12 patients, ORR and DCR in the non-clear cell histology were encouraging and consistent with data from ARON-1; nonetheless, these results should be considered exploratory and supportive of the ongoing further investigations in larger cohorts [19].

Predictably, PS-ECOG emerged as the strongest prognostic factor, and patients with PS-ECOG ≥ 2 had a significantly higher risk of progression and death. The pivotal trial included only patients with KPS ≥ 70, while PS data were not detailed in ARON-1. In a regional setting with documented higher mortality, performance status may also reflect social determinants of health and potential delays in diagnosis or treatment initiation. Ongoing prospective process evaluation within ONCOCAMP aims to correlate patient pathways with clinical outcomes. Grade ≥3 AEs were less frequent than reported in KEYNOTE-426 (14% vs. 30.5%). This lower rate may partly reflect underreporting in a retrospective real-world setting, particularly for low-grade, transient, or self-limiting toxicities, although improved toxicity management over the years and dose adjustments may also have contributed. No specific excess of treatment-related CNS toxicity was identified in the available records; however, CNS-specific complications, including intracranial hemorrhage, seizures, and corticosteroid use, were not systematically collected as dedicated variables; therefore, CNS-related safety findings should be interpreted cautiously.

Finally, we observed a higher incidence of grade 1–2 toxicity among female patients. Sex-related differences in ICI toxicity have been reported across multiple malignancies, with women in some studies experiencing a higher incidence of immune-related adverse events [20,21]. Several biological and pharmacological mechanisms have been proposed in the literature, including sex-related differences in immune activation and treatment response and pharmacokinetic or body-composition differences [22,23,24]. As our study did not include recording of sex-specific biomarkers or dedicated pharmacokinetic/immunological analyses, these mechanisms cannot be directly assessed. Accordingly, the observed association between female sex and low-grade toxicity should be considered exploratory, and retrospective reporting patterns, limited sample size, and unmeasured clinical factors may also have contributed to this finding. Future studies integrating immune, molecular, and liquid-biopsy biomarkers may help clarify the biological basis of interindividual differences in ICI–TKI tolerability and support more personalized toxicity monitoring [25,26,27].

This study has several limitations. First, its retrospective design inherently exposes the analysis to potential selection bias. Treatment allocation was not randomized and may have been influenced by clinical judgment, comorbidities, disease burden, or other factors not entirely accounted for in the available dataset. Second, the relatively limited sample size reduced statistical power, particularly for subgroup analyses. Follow-up duration, although consistent with real-world practice, may still be insufficient to observe mature survival endpoints, and the number of events was limited, particularly for OS. Moreover, some clinically relevant subgroups, including patients with ECOG PS ≥ 2 and non-clear-cell histology, were small. Therefore, multivariable hazard-ratio estimates may be unstable and should be interpreted as exploratory rather than definitive. Missing baseline covariate data were handled by complete-case analysis in Cox regression models, without imputation; this approach reduced the effective sample size of multivariable analyses and may have further limited statistical power. Finally, detailed information on the activity, prior local treatment, and stability of brain metastases at treatment initiation was not uniformly available, and no centralized radiologic review was performed.

Nevertheless, the consecutive inclusion of patients treated within a single, unified regional healthcare system enhances the internal consistency of clinical management and strengthens the representativeness of routine practice. While real-world data cannot establish treatment superiority in the absence of randomization, they provide valuable insight into treatment patterns, tolerability, and patient-related factors that may influence outcomes in daily clinical settings.

## 5. Conclusions

In this consecutive regional real-world cohort, first-line pembrolizumab plus axitinib showed clinically relevant disease control, with median PFS comparable to prior reports, although ORR was lower than expected. Performance status at diagnosis emerged as the strongest prognostic factor. Findings in non-clear-cell histology and potential sex-related differences in toxicity should be considered exploratory and warrant further investigation.

This study provides real-world evidence on treatment patterns and outcomes in an unselected network-based population. Future ROC/ONCOCAMP analyses should prospectively integrate clinical pathways, standardized radiologic assessment, pathology, molecular biomarkers, and, where feasible, multimodal data sources such as imaging, electronic health records, and omics data. In line with emerging AI-driven precision-oncology frameworks, such integrated approaches may further support patient stratification, biomarker discovery, and treatment selection [28].

## Figures and Tables

**Table 1:** Baseline characteristics.

	Overall
	(*N* = 117)
**Median age (range), years**	59 [34.0, 88.0]
Male, *n* (%)	84 (71.8%)
Female, *n* (%)	33 (28.2%)
**IMDC risk group, *n* (%)**	
Favorable	23 (19.6%)
Intermediate	55 (47.0%)
Poor	21 (18.0%)
Unknown	18 (15.4%)
**Comorbidities**	
Hypertension	50 (42.7%)
Cardiovascular disease	22 (18.8%)
Metabolic disease	17 (14.5%)
Diabetes	16 (13.7%)
Lung disease	4 (3.4%)
Hepatitis	3 (2.5%)
Autoimmune disease	3 (2.5%)
CKI	2 (1.7%)
**Previous nephrectomy, *n* (%)**	53 (45.3%)
**Histology**	
Clear cell	102 (87.2%)
Papillary	6 (5.1%)
Sarcomatoid features	4 (3.4%)
Chromophobe	1 (0.8%)
Mixed	1 (0.8%)
Unknown	3 (2.5%)
**Metastatic disease, *n* (%)**	
Recurrence	38 (32.5%)
*De novo*	79 (67.5%)
**Site of metastasis, *n* (%)**	
Brain	43 (36.8%)
Lung	41 (35.0%)
Bones	36 (30.8%)
Lymph nodes	34 (29.1%)
Adrenals	8 (6.8%)
Liver	7 (6.0%)
Pancreas	4 (3.4%)
Peritoneal carcinosis	3 (2.6%)
Spleen	2 (1.9%)
Rectum	2 (1.7%)
**Time from diagnosis to treatment <1 year *n* (%)**	77 (65.8%)
**BMI, median (range), kg/m^2^**	25.25 [18.75, 38.74]
**ECOG performance status**	
0	36 (30.8%)
1	56 (47.9%)
2	6 (5.1%)
3	1 (0.8%)
Unknown	18 (15.4%)

**Table 2:** Best response in the overall population and by histology subgroup.

	Overall (*n* = 117) *	Clear Cell (*n* = 102)	Non-Clear Cell (*n* = 12)
**ORR, *%(95% CI)***	27.3% (19.3% to 35.4%).	26.5% (17.9% to 35.0%)	41.7% (13.8% to 69.5%)
**DCR, *%(95% CI)***	79.5% (72.2% to 86.8%)	78.4% (70.4% to 86.4%)	83.3% (62.1% to 100%)
**CR, n (%)**	2 (1.8%)	2 (1.9%)	-
**PR, n (%)**	30 (25.6%)	25 (24.5%)	5 (41.7%)
**SD, n (%)**	61 (52.1%)	53 (52.0%)	5 (41.7%)
**PD, n (%)**	24 (20.5%)	22 (21.6%)	2 (16.7%)

* Missing histology data.

**Table 3:** Treatment-related adverse events.

Events	Grades 1, 2	Grades 3, 4
Number of Patients = 117		
**Mucosal inflammation**	9 (7.7%)	1 (0.8%)
**Hypertension**	11 (9.4%)	2 (1.7%)
**Hypotension**	3 (2.5%)	0
**Diarrhea**	28 (23.9%)	7 (6.0%)
**Hyperthyroidism**	2 (1.7%)	0
**Hypothyroidism**	15 (12.8%)	3 (2.5%)
**Asthenia**	21 (18.0%)	4 (3.4%)
**Hypertransaminasemia**	4 (3.4%)	3 (2.5%)
**Rash**	3 (2.5%)	0
**Weight decreased**	1 (0.8%)	0
**Myalgias**	2 (1.7%)	0
**Night sweats**	1 (0.8%)	0
**Weight loss**	1 (0.8%)	0
**Pyrexia**	1 (0.8%)	0
**Dyspnea**	1 (0.8%)	0
**Hepatitis**	0	1 (0.8%)
**Stomatitis**	1 (0.8%)	0
**Nausea**	6 (3.4%)	0
**Vomiting**	1 (0.8%)	0
**Decreased appetite**	3 (2.5%)	0
**Glossitis**	1 (0.8%)	0
**Dysgeusia**	1 (0.8%)	1 (0.8%)
**Hidradenitis suppurativa**	1 (0.8%)	0
**Rectal bleeding**	1 (0.8%)	0
**Increased ACTH**	1 (0.8%)	0
**Increased Cortisol**	1 (0.8%)	0
**Itching**	3 (2.5%)	0
**Anemia**	1 (0.8%)	0
**Autoimmune pneumonia**	0	2 (1.7%)
**Constipation**	2 (1.7%)	0
**Dysphonia**	1 (0.9%)	0
**Hyperbilirubinemia**	0	1 (0.8%)
**Hypertriglyceridemia**	2 (1.7%)	0
**HFS**	1 (0.8%)	0
**Ulcerative rectocolitis**	1 (0.8%)	0
**Embolism**	0	2 (1.7%)
**Intestinal perforation**	0	1 (0.8%)
**Vascular disease**	0	1 (0.8%)

**Table 4:** Comparison between ROC-RWD, the first interim analysis of the Keynote-426 trial results, and the ARON-1 study.

	ROC-RWD	KEYNOTE 426 (First Interim Analysis—2019)	ARON-1
**Sample size (n)**	117	432	607
**Follow up** Median months (IQR)	12.8 (0.5–33.6)	12.8 (0.1–22.0)	17 (15.9−68.1)
**Overall Survival** Median (95% CI)	Not reached	Not reached	52.2 (34.4−55.7)
**Progression-Free Survival** Median (95% CI)	15.1 (9.63-NA)	15.1 (12.6–17.7)	16.2 (14.2−20.2)
**Objective Response Rate** % (95% CI)	27.3%	59.3%	49%
**Duration of treatment** Median months (IQR)	10.8 (3.81–18.86)	10.4 (0.03–21.2)	NA
**Still receiving treatment** *N* (%)	52 (44%)	253 (59%)	NA
**Subsequent therapy** *N* (%)	27 (41%)	88 (50%)	206 (69%)

NA: not available.

## Data Availability

The original data are held by the National Cancer Institute, Fondazione IRCCS Pascale. The datasets generated and analyzed during the current study are available in the Zenodo repository at the following link: https://zenodo.org/uploads/15188363 (accessed on 3 February 2026). Further inquiries can be directed to the corresponding author.

## Supplementary Materials

The following supporting information can be downloaded at: https://www.mdpi.com/article/10.3390/curroncol33060325/s1, Table S1. Univariate analysis; Table S2. adjusted HRs for PFS; Table S3. adjusted HRs for OS.
