# Supplementary material for: Real-World Analysis of Metastatic Renal Cell Carcinoma Patients Treated with Pembrolizumab Plus Axitinib: Evidence from the Campania Oncology Network
Source: Curr Oncol. 2026 May 30;33(6):325. doi: 10.3390/curroncol33060325 (PMC13298080; doi:10.3390/curroncol33060325)
Supplement: Supplementary file 1 [file curroncol-33-00325-s001.zip › curroncol-4305480-supplementary.pdf]

Table S1. Univariate analysis.

Covariate	Median PFS (95% CI)	PFS (events)	*p value*	Median OS (95% CI)	OS (events)	*p value*
Gender Male	16.7 (9.4 , NA)	41/84	0.88	NA (NA, NA)	24/84	0.25
Female	13.4 (9.5, NA)	16/33		NA (9.5,NA)	13/33	
Age						0.16
<65 y.o.	16.7 (9.6, NA)	38/80	0.53	NA (NA, NA)	22/80	
>=65 y.o.	12.0 (7.4, NA)	19/37		19.1 (11.4, NA)	15/37	
Histology		51/102	0.77		34/102	0.51
Clear cell	12.9 (8.5, NA)	6/12		NA (NA, NA)	3/12	
Non-clear cell	16.7 (9.5 NA)			NA (13.1, NA)		
N. of organs with metastases at						
diagnosis	12.0 (8.5, NA)	20/38	0.54	16.2 (12.9, NA)	14/38	0.50
=1	16.7 (7.6, NA)	33/66		NA (NA, NA)	21/66	
>=2						
**PS ECOG**						
PS ECOG 0-1	13.4 (9.4, NA)	45/92	**0.001**	NA (19.1, NA)	29/92	**0.02**
PS ECOG 2-3	4.4 (1.4, NA)	7/7		9.5 (3.7, NA)	5/7	
IMDC risk score						
Favorable	NA (23.9, NA)	7/23	0.07	NA (NA, NA)	4/23	0.25
Intermediate	16.7 (9.4, NA)	26/55		NA (19.1, NA)	15/55	
Poor	12.9 (4.4, NA)	14/21		NA 12.9, NA)	9/21	

Table S2. adjusted HRs for PFS.

Characteristic	HR	n	95% CI	p-value
**ECOG**				
PS ECOG 0-1	—	45/92	—	
PS ECOG >=2	5.2	7/7	1.8, 14.4	0.001
**Age**				
<65 y.o.	—	38/80	—	
>=65 y.o	1.2	19/37	0.6, 2.4	0.6
**Gender**				
Female	—	16/33	—	
Male	1.3	41/84	0.7, 2.5	0.4
**IMDC risk score**				
Favourable	—	7/23	—	
Intermediate	1.1	26/55	0.4, 3.0	0.7
Poor	1.1	14/21	0.3, 3.2	0.9
**N. of metastasis at diagnosis**				
>=2	—	33/67	—	—
1	1.4	20/37	0.7, 2.9	0.3

[1] HR = Hazard Ratio, CI = Confidence Interval.

Table S3. adjusted HRs for OS.

Characteristic	HR	n	95% CI	p-value
**ECOG**				
PS ECOG 0-1<br>PS ECOG >=2	—<br>5.9	29/92<br>5/7	—<br>1.7, 20.4	 0.005
**Age**				
<65 y.o.<br>>=65 y.o	—<br>2.0	22/80<br>15/37	—<br>0.9, 4.7	 0.09
**Gender**				
Female<br>Male	—<br>0.9	13/33<br>24/84	—<br>0.4, 2.	 0.8
**IMDC risk score**				
Favorable	—	4/23	—	
Intermediate	0.9	15/55	0.2, 3.5	0.9
Poor	1.0	9/21	0.2, 3.5	0.9
**N. of metastasis at diagnosis**				
>=2<br>1	—<br>2.0	21/67<br>14/37	—<br>0.8, 5.0	—<br>0.1

HR = Hazard Ratio, CI = Confidence Interval.
